# Mandibular Metastatic Breast Cancer: A Rare Case Report with Diagnostic Challenge 

**DOI:** 10.30476/dentjods.2025.103764.2475

**Published:** 2025-09-01

**Authors:** Mohammad Mehdizadeh, Ali Lotfi, Saede Atarbashi-Moghadam, Parsa Eftekhari Moghadam

**Affiliations:** 1 Dept. of Oral and Maxillofacial Surgery, School of Dentistry, Qom University of Medical Sciences, Qom, Iran.; 2 Dept. of Oral and Maxillofacial Pathology, School of Dentistry, Shahid Beheshti University of Medical Sciences, Tehran, Iran.; 3 Undergraduate Student, Research Committee, School of Dentistry, Shahid Beheshti, University of Medical Sciences, Tehran, Iran.

**Keywords:** Metastasis, Breast cancer, Mandible, Oral cavity

## Abstract

Jawbone metastatic lesions are a diagnostic challenge because of their rarity and variable clinical, radiographic, and histopathologic characteristics. This paper presents a 57-year-old female with a chief complaint of lower face swelling. Cone beam computed tomography (CBCT) showed a multilocular radiolucency with right angle septa in the left mandibular area with cortical destruction. She had a history of right breast cancer about six years ago. Histopathologic examination revealed sheets of malignant small round cells. Immunohistochemistry (IHC) was only positive for cytokeratin (CK) and GATA3. CA15-3 tumor marker was higher than the normal range. Based on the aforementioned data, the diagnosis of metastatic breast carcinoma was performed. The whole-body and computed tomography (CT) scan showed just involvement in the left mandibular area. The radiographic appearance of metastatic lesions might be misleading, and microscopic sections might be poorly differentiated, therefore, a precise past medical history, IHC staining, and tumor markers are valuable issues in diagnosing oral cavity metastasis.

## Introduction

Metastatic lesions of the jaw are uncommon, with the most common primary sites being the breast in females and the lung in males [ 1 ]. Although it has been reported in a wide age range, it is most commonly seen in older people without a frank gender predilection [ 2 ]. 

Metastatic deposits have significant diagnostic and therapeutic importance in the head and neck, particularly in the oral and maxillofacial areas, because they can be the first sign of the spread of primary malignant cells from other organs [ 3 ].

The clinical signs and symptoms may vary, including pain, swelling, tenderness, paresthesia, pathologic fractures, and trismus. Radiographically, they show solitary or multiple radiolucencies with poorly defined, “motheaten” margins. Less commonly, mixed radiopaque/ radiolucent lesions can be detected, especially in metastatic prostate or breast adenocarcinomas with osteoblastic activity. The non-pathognomonic radiographic features often lead to incorrect interpretations of inflammatory processes [ 2 , 4 ]. In addition, they may not display typical clinical and radiographic manifestations of malignancy, making diagnosis challenging [ 1 ]. Here, we present a case of mandibular metastatic breast cancer with challenging radiographic and histopathologic features in a 57-year-old woman with a history of breast cancer.

## Case Presentation

**Figure 1:**
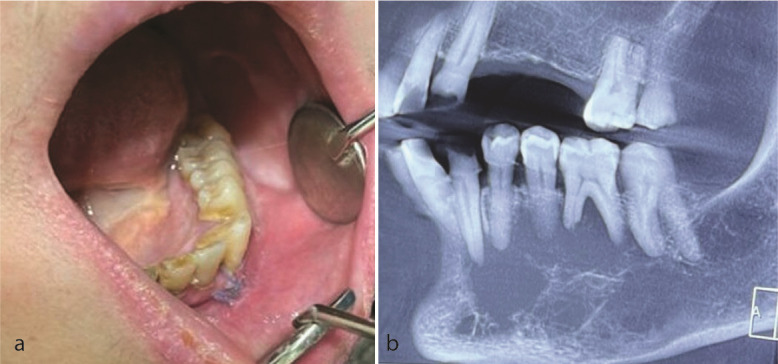
**a:** Photograph shows mild buco-lingual expansion of left mandible, **b:** Cone beam computed tomography (CBCT) displays a large lytic radiolucent lesion with right-angle septa

**Figure 2:**
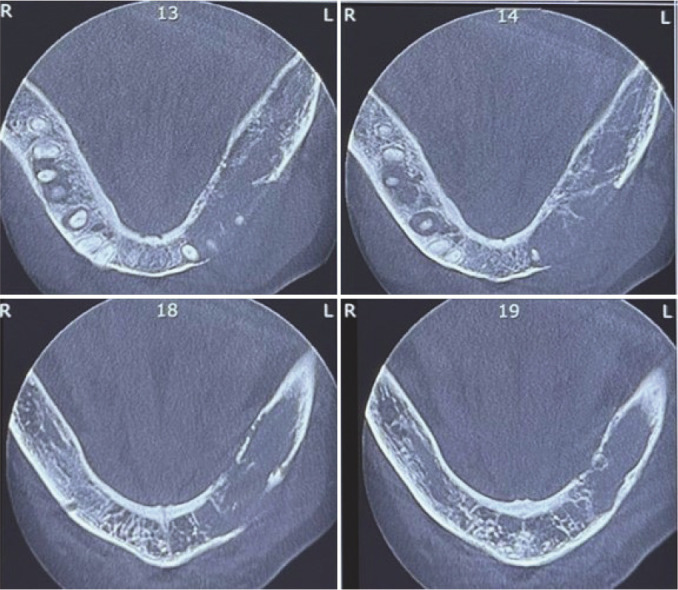
Axial views of the cone beam computed tomography reveal tumor extension and cortical destruction

**Figure 3:**
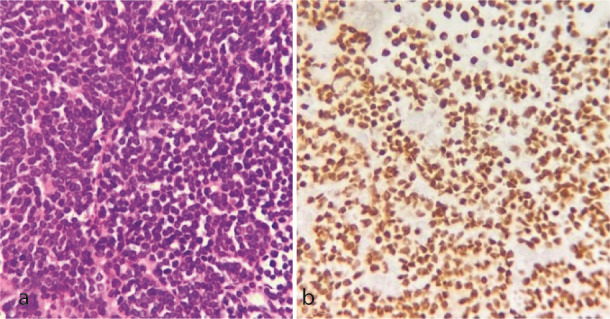
**a:** Histopathologic sections show a diffuse infiltration of small hyperchromatic round cells (hematoxylin and eosin staining (H & E), 400×); **b:** Immunohistochemistry (IHC) staining for GATA3 displays diffuse and strong positivity (IHC, 400×)

**Figure 4:**
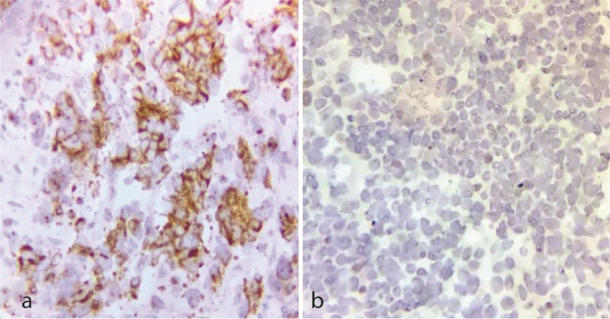
**a:** IHC staining for CK displays diffuse and strong positivity (IHC, 400×), **b:** IHC staining for ER was negative (IHC, 400×)

A 57-year-old woman with a chief complaint of left lo-were face swelling was referred to a private dental clinic (Qom, Iran) in October 2023. The intraoral examination revealed mild buccal and lingual expansion from the left lateral incisor extending to the second molar without any mucosal erosion or ulcer. There was no cervical lymphadenopathy (Figure 1a-b). Cone beam computed tomography (CBCT) showed a multilocular radiolucency from tooth 32 to 37 with right angle septa and cortical destruction. The extension of the lesion was more than its expansion (Figure 2). She had a history of right breast cancer in 2017 with a microscopic diagnosis of invasive ductal carcinoma, solid type, stage IIIc. She had undergone a wide surgical excision and chemo-radiotherapy. She took bisphosphonates and was under follow-up. Due to past medical history and radiographic features, a provisional diagnosis of odontogenic myxoma, metastatic cancer, and leukemia/lymphoma was made, and an incisional biopsy was performed under local anesthesia. Histopathologic examination showed a malignant neoplasm composed of sheets of small round cells with hyperchromatic nuclei and mitosis. Considering the medical history and microscopic features, an overall diagnosis of a small round cell tumor was rendered, and immunohistochemical (IHC) staining for cytokeratin (CK), leukocyte common antigen (LCA), CD-99, GATA3, estrogen (ER), progesterone (PR), and HER-2 was recommended. IHC was only positive for GATA3 and CK (Figure 3b, Figure 4a). Findings were consistent with metastatic carcinoma of breast origin. In whole-body scans and CT with and without contrast, no focus of involvement was seen except for the left mandibular area. Tumor markers such as CA15-3 (40.2u/ mL) were higher than the normal range (up to 31.3), but CEA (1.05ng/mL) and CA125 (12.2u/mL) were in the standard limits. Due to bone (mandible) metastasis, the patient was placed in stage IV. She was referred to an oncologist to complete the treatment. She has undergone two courses of chemotherapy and one course of radiotherapy, and after 13 months (November, 2024), the general condition of the patient was acceptable.

## Discussion

Bone is the preferred location of metastasis for many solid neoplasms [ 2 ]. According to Ho *et al*.'s study and literature review [ 2 ], the most common primary malignancies that metastasize to the jaw include lung, breast, kidney, bone, and colorectal. Moreover, based on Atarbashi-Moghadam *et al*. systematic review [ 5 ], the most common metastatic jaw tumors with bone origin are osteosarcoma and chondrosarcoma followed by Ewing sarcoma. Ewing sarcoma shows immunoreactivity with CD99 and NKX2.2. Furthermore, FLI1 or ERG may be found in patients harboring EWSR1:FLI1 or EWSR1: ERG mutations, respectively .

Table 1 shows brief information about the primary sites with IHC expression and gender predilection. The head and neck areas are not common sites of metastasis, and if detected, this deposition is frequently the result of secondary spread from other metastatic lesions, primarily lung cancers. Others have proposed that the metastatic deposit may originate directly from the primary organ site and bypass the lungs through the valveless vertebral venous plexus [ 8 ]. The current case also showed no focus on involvement except for the left mandibular area. Among the jaw bones, the mandible accounts for most of metastatic cases with a tendency to the molar areas [ 2 , 4 - 5 , 8 ]. The pattern of metastatic spread of cancer is not by chance. The interaction between the surface receptors of spreading tumoral cells and the endothelial receptors of the target organ leads to the selection of the site of metastasis [ 8 ] Oral and maxillofacial metastases may be challenging to detect on routine radiographic examination, especially in the early stages [ 8 ]. Some previous reports have noted that the radiographic appearance of the metastatic lesion may not show signs in favor of malignancy [ 1 ]. Watkins *et al*. [ 1 ] reported a case that showed similar features of an odontogenic cyst with an impacted tooth. The most common presentation of metastatic lesions is ill-defined osteolytic change with root resorption, cortical destruction, and soft tissue infiltration [ 1 - 2 , 8 ]. Nel *et al*. [ 8 ] found that cortical destruction was the most common radiological finding, with bone expansion seen in a small number of patients. In the current patient, the radiographic features evoked some characteristics of odontogenic myxoma such as right angle septa and a tendency to extension in the jaw bone. A proper and accurate past medical and family history is beneficial in diagnosing metastasis [ 1 , 4 ]. In the present case, the microscopic appearance was poorly differentiated (round cell tumor) and did not suggest carcinoma. Taking a detailed past medical history helped us choose the precise immunomarkers and determine the origin of tumors.Depending on the microscopic features of the neoplasm, immunoreactivity against pan cytokeratins, p63, S100, vimentin or desmin, and LCA (CD45) typically comprise the preliminary screening panel to rule out metastatic carcinoma, melanoma, sarcoma, and lymphoma, respectively [ 2 , 9 ]. For example, a CK7+/CK20 - immune profile with TTF1 positivity would indicate lung primary, while a CK7-/CK20+, CDX2+, SATB2+ proposes colorectal origin. CK7, ER/PR, GATA3, and mammaglobin help detect metastatic adenocarcinomas of the breast, while NKX3.1 accompanied by other prostate-restricted markers, such as PSA or PSAP, can help determine prostatic origin. Negative CK7/CK20 with positive RCC, PAX8, and CD10 would suggest renal metastasis. A CK7+/CK20-, WT1+, and PAX8+ immune profile propose an ovarian origin [ 2 , 4 ]. Tumor markers such as CA15-3 may be supportive in determining the extent of the spread of breast cancer, and high CA15-3 can be an important prognostic factor for the presence of bone metastasis in these patients [ 10 ]. The management of oral metastatic breast carcinomas is principally palliative and may comprise radiotherapy, chemotherapy, hormone therapy, and seldom surgery [ 11 ]. It is necessary to evaluate the menopausal status and the expression of ER, PR, and HER2 to define the treatment strategy for metastatic breast cancer. HER2 overexpression is related to poorer clinical course and survival rate. Moreover, hormone therapy depends on ER and PR status [ 12 ]. In the present case, HER-2 (+1), ER, and PR were negative. In general, mandibular metastatic carcinoma shows a poor prognosis, and most patients die within one year, whereas the 4-year survival rate was reported to be 10% [ 13 ]. 

**Table 1:** The most common primary sites that have metastasized to the jaws

Most common primary site	Gender predilection	IHC staining based on origin
Lung	M>F	Positive: TTF-1, CK7
		Negative: CK20
Breast	F>M	Positive: CK7, ER/PR, GATA3, mammaglobinKidney
Kidney	M>F	Positive: RCC, PAX8, and CD10
		Negative: CK7, CK20
Bone (osteosarcoma, chondrosarcoma and Ewing sarcoma)	M>F	Ewing sarcoma:
		Positive: CD99, Fli-1 and ERG
Colorectal	F>M	Positive: CK20, CDX2, SATB2
		Negative: CK7

Informed consent was obtained from the patient for publishing her clinical photograph and radiography. 

## Conclusion

The radiographic appearance of metastatic lesions may be deceiving and histopathologic sections may be poorly differentiated, therefore, the origin of the primary malignancy cannot be determined precisely. Consequently, a detailed past medical history, IHC staining, and tumor markers are practical in diagnosing the origin of metastatic lesions.
